# Preoperative serum lipids as prognostic predictors in esophageal squamous cell carcinoma patients with esophagectomy

**DOI:** 10.18632/oncotarget.15651

**Published:** 2017-02-23

**Authors:** Pengxiang Chen, Lihui Han, Cong Wang, Yibin Jia, Qingxu Song, Jianbo Wang, Shanghui Guan, Bingxu Tan, Bowen Liu, Wenqiao Jia, Jianfeng Cui, Wei Zhou, Yufeng Cheng

**Affiliations:** ^1^ Department of Radiation Oncology, Qilu Hospital of Shandong University, Jinan, People’s Republic of China; ^2^ Department of Urology, Qilu Hospital of Shandong University, Jinan, People’s Republic of China

**Keywords:** esophageal cancer, squamous cell carcinoma, serum lipids, prognosis

## Abstract

This study was to evaluate the prognostic significance of serum lipids in esophageal squamous cell carcinoma patients who underwent esophagectomy. Preoperative serum lipids were collected from 214 patients who were diagnosed with esophageal squamous cell carcinoma. All of the patients received esophagectomy in Qilu Hospital of Shandong University from January 2007 to December 2008. The records and data were analyzed retrospectively. We found that low total cholesterol (for T stage, *p* = 0.006; for TNM stage, *p* = 0.039) and low-density lipoprotein cholesterol (for T stage, *p* = 0.031; for TNM stage, *p* = 0.035) were associated with advanced T stage and TNM stage. Kaplan-Meier survival analysis indicated that low total cholesterol and low-density lipoprotein cholesterol were associated with shorter disease-free survival(for total cholesterol, *p* = 0.045; for low-density lipoprotein cholesterol, *p* < 0.001) and overall survival (for total cholesterol, *p* = 0.043; for low-density lipoprotein cholesterol, *p* < 0.001). Lower low-density lipoprotein cholesterol/high-density lipoprotein cholesterol ratio (LHR) indicated poorer disease-free survival and overall survival (both *p* < 0.001). In the multivariate analysis, low-density lipoprotein cholesterol and LHR were independent prognostic factors for disease-free survival and overall survival. In conclusion, our study indicated that preoperative serum total cholesterol and low-density lipoprotein cholesterol are prognostic factors for esophageal squamous cell carcinoma patients who underwent esophagectomy. LHR can serve as a promising serum lipids-based prognostic indicator.

## INTRODUCTION

In 2012, there were 455 800 new esophageal cancer (EC) cases and 400 200 deaths worldwide, especially in developing countries [1]. In high-risk region, including some Asian countries, esophageal squamous cell carcinoma (ESCC) were responsible for 90% of all cases with a significant increasing incidence rate [2]. According to the National Comprehensive Cancer Network (NCCN) guideline of ESCC (version 2, 2016), esophageal resection should be considered for all physiologically fit patients with resectable esophageal cancer [3]. Surgical treatment is still the recommended curative option in ESCC patients considering multiple anti-tumor modalities, unless contraindications exist. Nevertheless, the 5-year overall survival (OS) remains only 26.2% to 49.4% after surgery [4]. Therefore, there is an urgent need for verifying effective biomarkers to predict prognosis of ESCC patients after surgery.

Various studies had discussed the close relationship between serum lipids and cancer, including gastrointestinal cancer, breast cancer and other cancers [5–8]. Yan-Yan Liu et al found that pretreatment high-density lipoprotein cholesterol (HDL-C) was a poor prognostic factor in nasopharyngeal carcinoma patients by enhancing proliferation and migration of tumor cells [9]. It is broadly accepted that low total cholesterol (TC) and low-density lipoprotein cholesterol (LDL-C) are highly relevant with malignant tumors, especially tumors of digestive system [8, 10, 11]. Saito et al found low LDL-C was associated with higher mortality, being a prognostic biomarker in liver patients [12]. As reported, serum TC and LDL-C significantly decreased in EC patients, indicating an altered lipid metabolism associated with EC [13]. Considering disequilibrium of lipids metabolism commonly occurred in cancer patients, the indicator containing several lipids may reflect the change of serum lipids more accurately and comprehensively. One research reported that LDL-C and high-density lipoprotein cholesterol (HDL-C) ratio (LHR) was a prognostic factor in colorectal patients, providing further prognositic information than single LDL-C or HDL-C [5].

Although serum lipids have been discussed in various cancers, studies about lipids and ESCC are limited. Relationships of serum lipids with survival prognosis in ESCC patients are also unclear. To our best knowledge, no research has been published previously regarding the LHR in ESCC patients. Consequently, we conducted this retrospective study to verify the correlations of serum lipids with clinicopathological characteristics and assess their prognostic values on disease-free survival (DFS) and OS in ESCC patients.

## RESULTS

### Clinicopathological characteristics of patients

Clinicopathological characteristics for 214 esophageal squamous carcinoma patients were shown in Table 1. Our research included 42 (19.6%) female and 172 (80.4%) male. And the median age was 60 years, ranging from 32 to 84 years. 114 (53.3%) patients’ tumor location was in the middle third of esophagus while 76 (35.5%) patients’ location occurred in the lower third, and only 24 (11.2%) patients’ tumor was located in the upper third. 128 (59.9%) patients received surgery alone, and adjuvant therapy were adopted among 86 (40.1%) patients, including postoperative radiotherapy in 39 patients, postoperative chemotherapy in 15 patients and postoperative chemoradiotherapy in 32 patients. The median follow-up time was 38.8 months (range 1.3–71.0 months). During the follow-up period, recurrence occurred in 101 (47.2%) patients, in which 12 cases had surgical anastomosis recurrences, 5 cases had local recurrences, 40 cases had lymph nodes metastasis and 44 cases developed distant metastasis.

**Table 1:** Clinical and histopathological characteristics of 214 esophageal squamous cell carcinoma patients

Characteristic		Patients, *n*(%)
Gender	Female/male	42 (19.6%)/172 (80.4%)
Ages (years)	Mean±SD	60.69±8.55
	Median (range)	60 (32-84)
Tumor length (cm)	<4	86 (40.2%)
	≥4	128 (59.8%)
Tumor location	Upper	24 (11.2%)
	Middle	114 (53.3%)
	Lower	76 (35.5%)
Differential grade	Well	54 (25.2%)
	Middle	90 (42.1%)
	Poor	70 (32.7%)
T stage	T1	22 (10.3%)
	T2	60 (28.0%)
	T3	118 (55.1%)
	T4	14 (6.6%)
N stage	N0	126 (58.9%)
	N1-3	88 (41.1%)
TNM stage	I	28 (13.1%)
	II	107 (50.0%)
	III	79 (36.9%)
Adjuvant therapy	No	128 (59.9%)
	Radiotherapy	39 (18.2%)
	Chemotherapy	15 (7.0%)
	Chemoradiotherapy	32 (14.9%)
Recurrence	Yes	101 (47.2%)
	No	113 (52.8%)
TG (mmol/L)	Mean±SD	1.05±0.48
	Median (range)	0.95 (0.38-3.78)
TC (mmol/L)	Mean±SD	4.67±0.94
	Median (range)	4.62 (1.92-7.72)
LDL-C (mmol/L)	Mean±SD	3.06±0.84
	Median (range)	2.99 (1.00-6.70)
HDL-C (mmol/L)	Mean±SD	1.49±0.48
	Median (range)	1.43 (0.71-4.02)
LHR	Mean±SD	2.23±0.85
	Median (range)	2.11 (0.38-6.03)

Abbreviations: RT: radiotherapy CT: chemotherapy CRT: chemoradiotherapy TG: triglyceride TC: total cholesterol LDL-C: low-density lipoprotein cholesterol HDL-C: high-density lipoprotein cholesterol LHR: LDL-C/HDL-C ratio SD: standard deviation

### Associations of TG, TC, LDL-C and HDL-C with clinicopathological characteristics

Data about associations of TG, TC, LDL-C and HDL-C with clinicopathological characteristics were shown in Tables 2 & 3. In ESCC patients, TC (for T stage, *p* = 0.006; for TNM stage, *p* = 0.039) and LDL-C (for T stage, *p* = 0.031; for TNM stage, *p* = 0.035) decreased with both advanced T stage and TNM stage while TG and HDL-C showed no such trends. All the lipids exhibited negative association with differential grade and N stage.

**Table 2:** Clinical status and TG, TC, LDL-C and HDL-C in esophageal squamous cell carcinoma

Characteristics	TG (mmol/L)		TC (mmol/L)		LDL-C (mmol/L)		HDL-C (mmol/L)	
	Mean	SD	Mean	SD	Mean	SD	Mean	SD
Differential grade								
Well	1.02	0.36	4.62	0.83	3.03	0.79	1.43	0.35
Middle	0.99	0.46	4.67	0.80	3.01	0.74	1.47	0.42
Poor	1.15	0.58	4.77	0.99	3.14	1.01	1.57	0.63
*p* value	0.238		0.834		0.769		0.424	
T stage								
T1	1.07	0.52	5.25	1.02	3.47	0.92	1.52	0.36
T2	1.10	0.67	4.74	0.79	3.16	0.75	1.54	0.44
T3	1.03	0.36	4.59	0.86	2.94	0.87	1.48	0.53
T4	0.98	0.40	4.41	0.72	2.90	0.74	1.32	0.32
*p* value	0.791		0.006*		0.031*		0.503	
N stage								
N0	1.07	0.51	4.77	0.84	3.07	0.82	1.51	0.43
N1-3	1.02	0.48	4.58	0.91	3.00	0.88	1.47	0.55
*p* value	0.446		0.111		0.501		0.519	
TNM stage								
I	1.02	0.46	5.05	1.04	3.44	0.84	1.49	0.36
II	1.09	0.55	4.68	0.77	2.99	0.77	1.50	0.44
III	1.01	0.38	4.56	0.91	3.00	0.92	1.48	0.57
*p* value	0.496		0.039*		0.035*		0.952	

Abbreviations: TG: triglyceride TC: total cholesterol LDL-C: low-density lipoprotein cholesterol

HDL-C: high-density lipoprotein cholesterol SD: standard deviation

**Table 3:** The characteristics of 214 esophageal squamous cell carcinoma patients grouped by TC and LDL-C

Characteristics		TC (mmol/L)		*p* value	LDL-C (mmol/L)		*p* value
		<4.79 (*N*= 131)	≥4.79 (*N*= 83)		<3.23 (*N*= 134)	≥3.23 (*N*= 80)	
Gender	Female	18	24	0.006*	24	18	0.413
	Male	113	59		110	62	
Ages (years)	<60	63	33	0.232	65	31	0.165
	≥60	68	50		69	49	
Tumor length (cm)	<4	50	36	0.449	52	34	0.594
	≥4	81	47		82	46	
Tumor location	Upper	14	10	0.594	15	9	0.983
	Middle	67	47		72	42	
	Lower	50	26		47	29	
Differential grade	Well	35	19	0.663	34	20	0.968
	Middle	56	34		57	33	
	Poor	40	30		43	27	
T stage	T1	10	12	0.200	9	13	0.044*
	T2	35	25		34	26	
	T3	75	43		81	37	
	T4	11	3		10	4	
N stage	N0	73	53	0.239	73	53	0.090
	N1-3	58	30		61	27	
TNM stage	I	14	14	0.257	11	17	0.023*
	II	64	43		70	37	
	III	53	26		53	26	
Adjuvant therapy	No	74	54	0.136	78	50	0.147
	Radiotherapy	26	13		26	13	
	Chemotherapy	13	2		13	2	
	Chemoradiotherapy	18	14		17	15	
Recurrence	Yes	60	41	0.608	67	34	0.288
	No	71	42		67	46	

Abbreviations: TC: total cholesterol LDL-C: low-density lipoprotein cholesterol RT: radiotherapy CT: chemotherapy CRT: chemoradiotherapy

By adopting ROC analysis, we obtained the optimal cutoff values of 4.79 mmol/L for TC and 3.23 mmol/L for LDL-C (TG and HDL-C showed negative results, so the data was not given). Relied on the cutoff values, 131 cases (61.2%) were assigned into low TC group and 83 (38.8%) cases were assigned into high TC group. 134 cases (62.6%) were included in low LDL-C group while 80 cases (37.4%) were included in high LDL-C group. In Table 3, low TC was only significantly associated with gender male (*p* = 0.006). Differences were discernable in regard to T stage (*p* = 0.044) and TNM stage (*p* = 0.023) between two groups of LDL-C.

### Survival and evaluation of the prognostic value of TC, LDL-C and LHR

**Figure 1:**
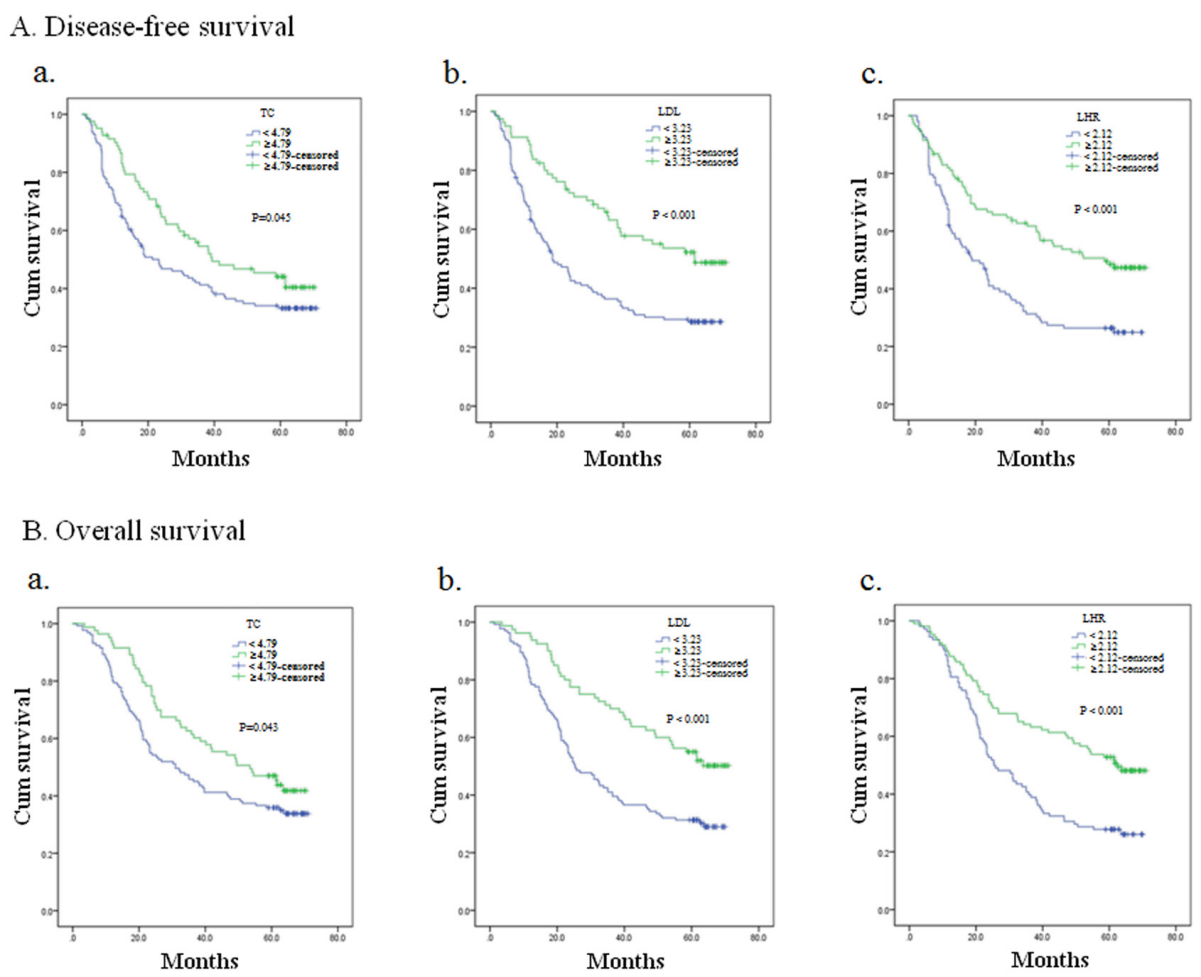
Kaplan–Meier analysis for disease-free survival and overall survival of esophageal squamous cell carcinomapatients based on preoperative TC, LDL-C and LDL-C/HDL-C ratio (LHR) at the end of follow-up

In our research, the median DFS was 29.8 months and 5-year DFS was 32.7%. The median OS was 38.8 months and 5-year OS was 36.4%. Kaplan–Meier curves of DFS and OS based on TC and LDL-C were given in Figure 1 (TG and HDL-C had no relationship with survival in our analysis, so the data was not shown ). The curves implied that patients with high TC and LDL-C obviously got a trend towards superior DFS (for TC, *p* = 0.045; for LDL-C, *p* < 0.001) and OS (for TC, *p* = 0.043; for LDL-C, p < 0.001). Considering serum lipids were related to nutritional condition, we analyzed DFS and OS in patients with normal pretreatment serum albumin (Table 4). Patients with lower TC and LDL-C showed inferior DFS (for TC, *p* = 0.034; for LDL-C, *p* < 0.001) and OS (for TC, *p* = 0.024; for LDL-C, *p* < 0.001).

**Table 4:** DFS and OS of esophageal squamous cell carcinoma patients with normal pretreatment serum albumin according to their TC and LDL-C

Variable		Patients	DFS Median(range)	*p* value	OS Median(range)	*p* value
TC (mmol/L)	<4.79	92	18.6 (1.3-71.0)	0.034*	31.6(1.3-71.0)	0.024*
	≥4.79	71	38.3 (1.5-70.4)		54.5(3.5-70.4)	
LDL-C (mmol/L)	<3.23	97	17.7(1.3-69.6)	<0.001*	25.8(1.3-69.9)	<0.001*
	≥3.23	66	55.3(1.5-71.0)		60.4(3.5-71.0)	

Abbreviations: TC: total cholesterol LDL-C: low-density lipoprotein cholesterol DFS: disease-free survival OS: overall survival

LHR was analyzed in this study and its optimal cutoff value was determined as 2.12. Kaplan–Meier curves of DFS and OS based on LHR was given in Figure 1. Patients with low LHR showed poor DFS and OS (both *p* < 0.001). To evaluate whether the relationship between LHR and survival was merely affected by elevated LDL-C, patients were stratified according to their LDL-C levels (Table 5). In patients with no elevations in LDL-C, those who had lower LHR tended to show inferior DFS (*p* = 0.036) and OS (*p* = 0.044). In patients with elevated LDL-C, LHR showed no association with DFS (*p* = 0.071) and OS (*p* = 0.065).

**Table 5:** DFS and OS of esophageal squamous cell carcinoma patients with elevations and no elevations in pretreatment serum LDL-C according to their LHR

		LHR		*p* value
		<2.12	≥2.12	
Patients with elevations in LDL-C				
	Patients, n (%)	17 (25.4%)	50 (74.6%)	
	DFS Median (range)	29.0 (6.0-69.6)	59.7 (1.5-71.0)	0.071
	OS Median (range)	40.2 (12.0-69.9)	61.4 (3.5-71.0)	0.065
Patients with no elevations in LDL-C				
	Patients, n (%)	92 (61.5%)	55 (38.5%)	
	DFS Median (range)	17.2 (1.3-69.6)	29.4 (1.3-69.4)	0.036*
	OS Median (range)	24.2 (3.0-69.9)	47.5 (1.3-69.4)	0.044*

Abbreviations: LDL-C: low-density lipoprotein cholesterol LHR: LDL-C/HDL-C ratio

DFS: disease-free survival OS: overall survival

### Univariate and multivariate survival analyses

The results of univariable Cox regression analysis related to DFS and OS were shown in Table 6. Various factors were associated with DFS and OS, including differential grade, T stage, N stage, TNM stage, TC, LDL-C, LHR and recurrence (all *p* < 0.05). Considering LDL-C is a component of LHR, two multivariate analyses consisting of LDL-C or LHR were operated, respectively. As Table 7 showed, LDL-C (*p* = 0.008; hazard ratio (HR), 0.543; 95% confidence interval (CI), 0.349-0.843), LHR (*p* = 0.004; HR, 0.567; 95% CI, 0.395-0.815) and N stage (*p* < 0.001) were independent prognostic factors for DFS of ESCC patients. As to OS, LDL-C (*p* = 0.010; HR, 0.562; 95% CI, 0.360-0.875), LHR ( *p* = 0.020; HR, 0.658; 95% CI, 0.457-0.948), N stage (*p* = 0.007) and recurrence (*p* < 0.001) were independent prognostic factors in ESCC patients.

**Table 6:** Univariate analysis of survival of esophageal squamous cell carcinoma treated by surgery

Characteristic	DFS			OS		
	*p* value	HR	95% CI	*p* value	HR	95% CI
Female	0.711	0.921	0.596-1.424	0.705	0.920	0.600-1.413
Age <60 (years)	0.853	1.033	0.732-1.457	0.848	0.967	0.687-1.362
Tumor length <4 (cm)	0.311	1.199	0.844-1.702	0.307	1.199	0.846-1.699
Tumor location						
Upper		1.000	Ref.		1.000	Ref.
Middle	0.470	0.814	0.465-1.423	0.45	0.806	0.461-1.410
Lower	0.737	0.905	0.507-1.617	0.655	0.876	0.491-1.564
Differential grade						
Well		1.000	Ref.		1.000	Ref.
Middle	0.290	1.281	0.810-2.025	0.301	1.273	0.806-2.009
Poor	0.001*	2.130	1.340-3.385	0.003*	2.016	1.270-3.200
T stage						
T1		1.000	Ref.		1.000	Ref.
T2	0.078	2.003	0.925-4.338	0.100	1.913	0.883-4.143
T3	0.003*	2.992	1.444-6.197	0.004*	2.905	1.403-6.015
T4	0.002*	4.156	1.692-10.206	0.001*	4.410	1.797-10.821
N stage:N0/N1-3	<0.001*	2.607	1.841-3.692	<0.001*	2.475	1.756-3.489
TNM stage						
I		1.000	Ref.		1.000	Ref.
II	0.025*	2.235	1.107-4.513	0.026*	2.214	1.097-4.467
III	<0.001*	5.052	2.505-10.186	<0.001*	4.881	2.424-9.828
Adjuvant therapy						
No		1.000	Ref.		1.000	Ref.
Radiotherapy	0.234	1.302	0.843-2.012	0.271	1.277	0.826-1.974
Chemotherapy	0.833	0.928	0.465-1.854	0.896	0.955	0.478-1.907
Chemoradiotherapy	0.482	1.190	0.733-1.932	0.285	1.303	0.802-2.115
TG ≥1.20 mmol/l	0.142	1.312	0.913-1.885	0.148	1.306	0.910-1.875
TC ≥4.79 mmol/l	0.046*	0.670	0.480-0.986	0.045*	0.695	0.487-0.992
LDL-C ≥3.23 mmol/l	<0.001*	0.503	0.346-0.732	<0.001*	0.496	0.341-0.722
HDL-C ≥1.10 mmol/l	0.352	0.820	0.540-1.245	0.379	0.829	0.546-1.258
LHR ≥2.12 mmol/l	<0.001*	0.519	0.366-0.735	<0.001*	0.526	0.371-0.745
Recurrence: yes/no	n.d.	n.d.	n.d.	<0.001*	0.238	0.164-0.347

Abbreviations: RT: radiotherapy CT: chemotherapy CRT: chemoradiotherapy TG: triglyceride TC: total cholesterol LDL-C: low-density lipoprotein cholesterol HDL-C: high-density lipoprotein cholesterol LHR: LDL-C/HDL-C ratio DFS: disease-free survival OS: overall survival HR: hazard ratio 95% CI: 95% confidential interval n.d.: not done Ref.: Reference

**Table 7:** Multivariate analysis of survival of esophageal squamous cell carcinoma treated by surgery

Characteristic	DFS			OS		
	*p* value	HR	95% CI	*p* value	HR	95% CI
(a) Analysis including LDL-C (omitting LHR)						
Differential grade						
Well		1.000	Ref.		1.000	Ref.
Middle	0.747	1.081	0.673-1.736	0.582	1.145	0.708-1.852
Poor	0.123	1.494	9.897-2.489	0.456	1.215	0.729-2.025
T stage						
T1		1.000	Ref.		1.000	Ref.
T2	0.430	1.370	0.627-2.995	0.606	1.233	0.556-2.731
T3	0.100	1.864	0.888-3.912	0.142	1.759	0.828-3.734
T4	0.087	2.230	0.889-5.594	0.216	1.801	0.710-4.571
N stage: N0/N1-3	<0.001*	2.047	1.385-3.023	0.008*	1.687	1.145-2.485
TC ≥4.79 mmol/l	0.743	1.073	0.705-1.634	0.623	0.900	0.590-1.372
LDL-C ≥3.23 mmol/l	0.008*	0.543	0.349-0.843	0.010*	0.562	0.360-0.875
Recurrence: yes/no	n.d.	n.d.	n.d.	<0.001*	0.266	0.180-0.393
(b) Analysis including LHR (omitting LDL-C)						
Differential grade						
Well		1.000	Ref.		1.000	Ref.
Middle	0.978	1.007	0.623-1.627			
Poor	0.219	1.380	0.826-2.305	0.457	1.213	0.729-2.018
T stage						
T1		1.000	Ref.		1.000	Ref.
T2	0.806	1.106	0.495-2.469	0.772	1.127	0.501-2.536
T3	0.162	1.708	0.806-3.620	0.189	1.667	0.778-3.574
T4	0.149	1.987	0.783-5.045	0.297	1.653	0.643-4.247
N stage: N0/N1-3	<0.001*	2.100	1.422-3.101	0.007*	1.702	1.158-2.501
TC ≥4.79 mmol/l	0.414	0.857	0.592-1.241	0.086	0.720	0.495-1.047
LHR ≥2.12 mmol/l	0.004*	0.567	0.395-0.815	0.020*	0.658	0.457-0.948
Recurrence: yes/no	n.d.	n.d.	n.d.	<0.001	0.278	0.188-0.410

Abbreviations: TC: total cholesterol LDL-C: low-density lipoprotein cholesterol LHR: LDL-C/HDL-C ratio DFS: disease-free survival OS: overall survival HR: hazard ratio 95% CI: 95% confidential interval n.d.: not done Ref.: Reference

### Subgroup analysis by postoperative adjuvant treatment

**Figure 2:**
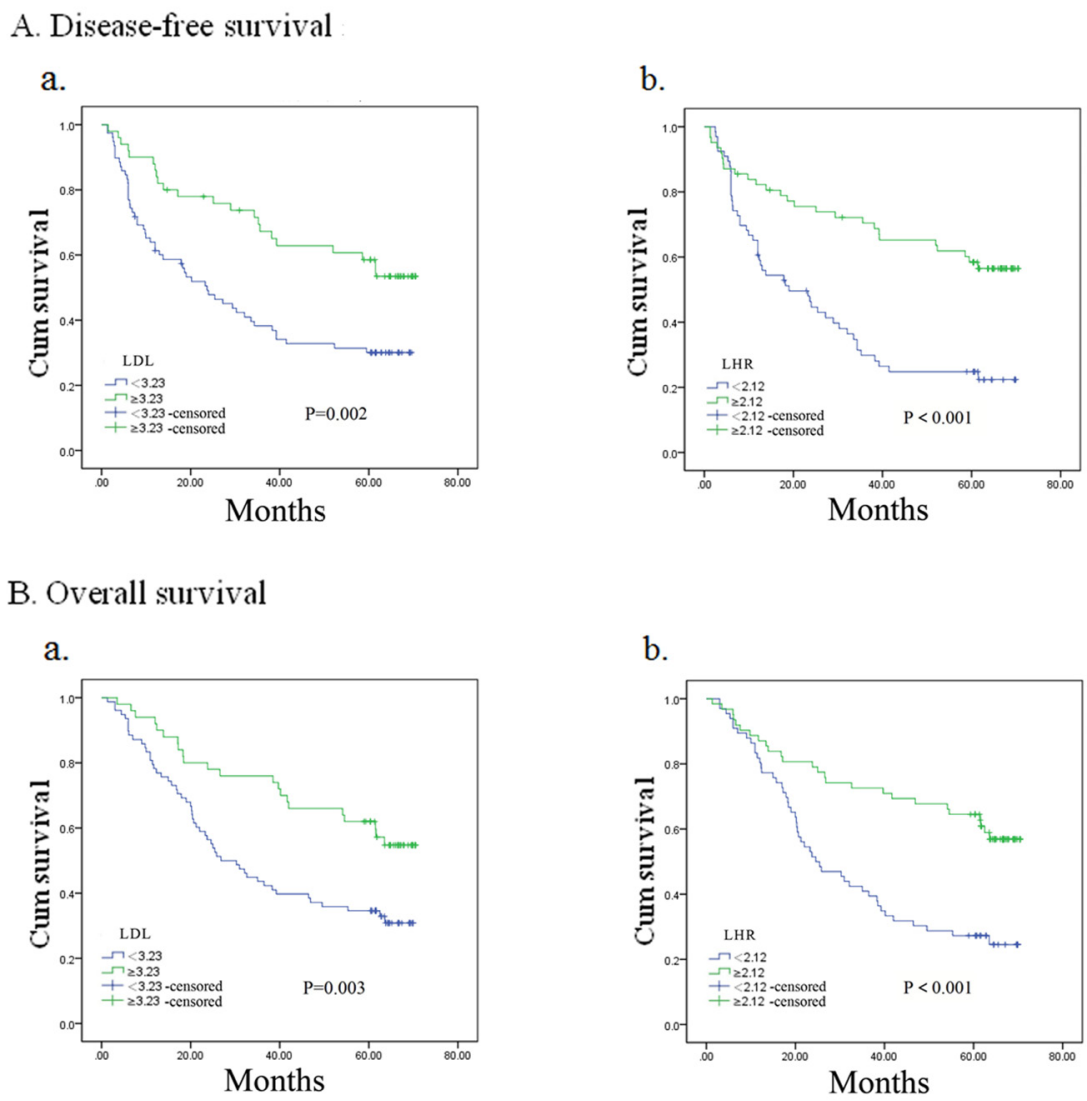
Kaplan–Meier analysis for disease-free survival and overall survival in esophageal squamous cell carcinoma patients without adjuvant treatment according to preoperative LDL-C, LDL-C/HDL-C ratio (LHR)

**Figure 3:**
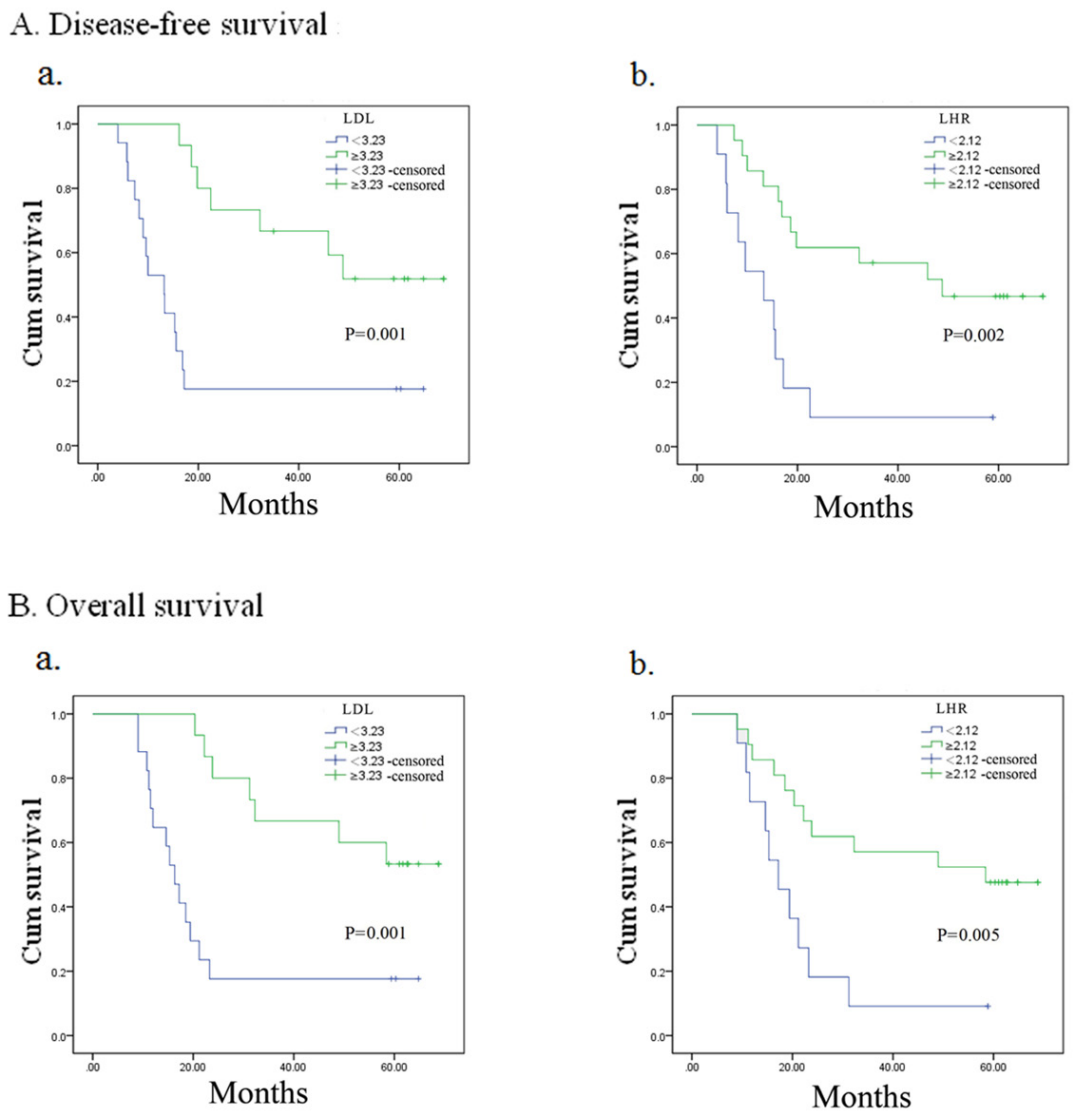
Kaplan–Meier analysis for disease-free survival and overall survival in esophageal squamous cell carcinoma patients with adjuvant chemoradiotherapy according to preoperative LDL-C, LDL-C/HDL-C ratio (LHR)

For ESCC patients without postoperative adjuvant treatment, Kaplan–Meier analysis implied that LDL-C and LHR were both prognostic predictive factors for DFS (for LDL-C, *p* = 0.002; for LHR, *p* < 0.001) and OS (for LDL-C, *p* = 0.003; for LHR, *p* < 0.001) (Figure 2). LDL-C and LHR were not associated with DFS and OS when patients received postoperative adjuvant radiotherapy or chemotherapy (all *p* > 0.05) (the figures were not shown). However, DFS (for LDL-C, *p* = 0.001; for LHR, *p* = 0.002) and OS (for LDL-C, *p* = 0.001; for LHR, *p* = 0.005) differed significantly in patients who adopted postoperative adjuvant chemoradiotherapy according to LDL-C or LHR (Figure 3).

### Subgroup analysis by pathological stage

**Figure 4:**
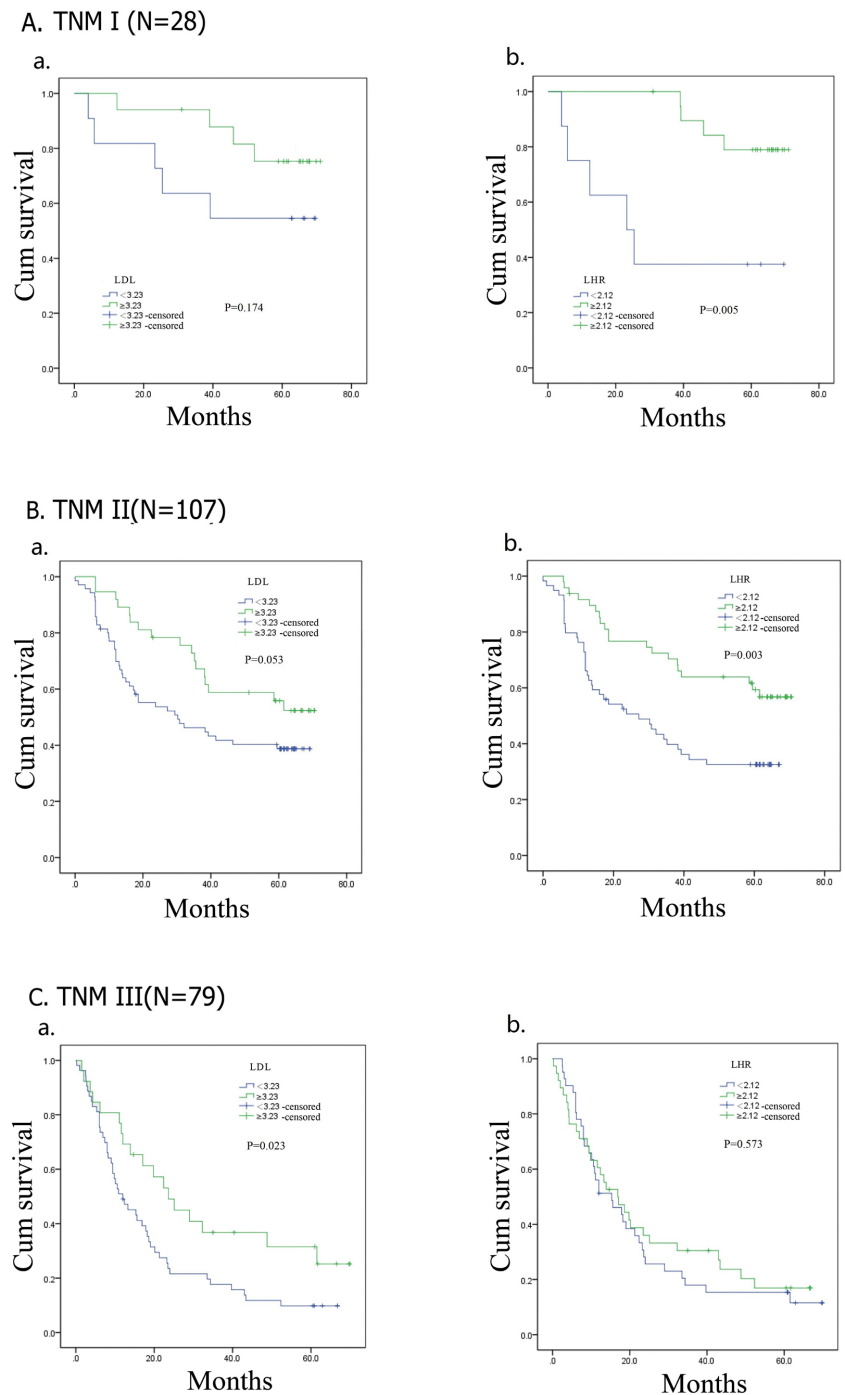
Kaplan–Meier analysis for disease-free survival of TNM I, II, III esophageal squamous cell carcinoma patients according to preoperative LDL-C, LDL-C/HDL-C ratio (LHR)

**Figure 5:**
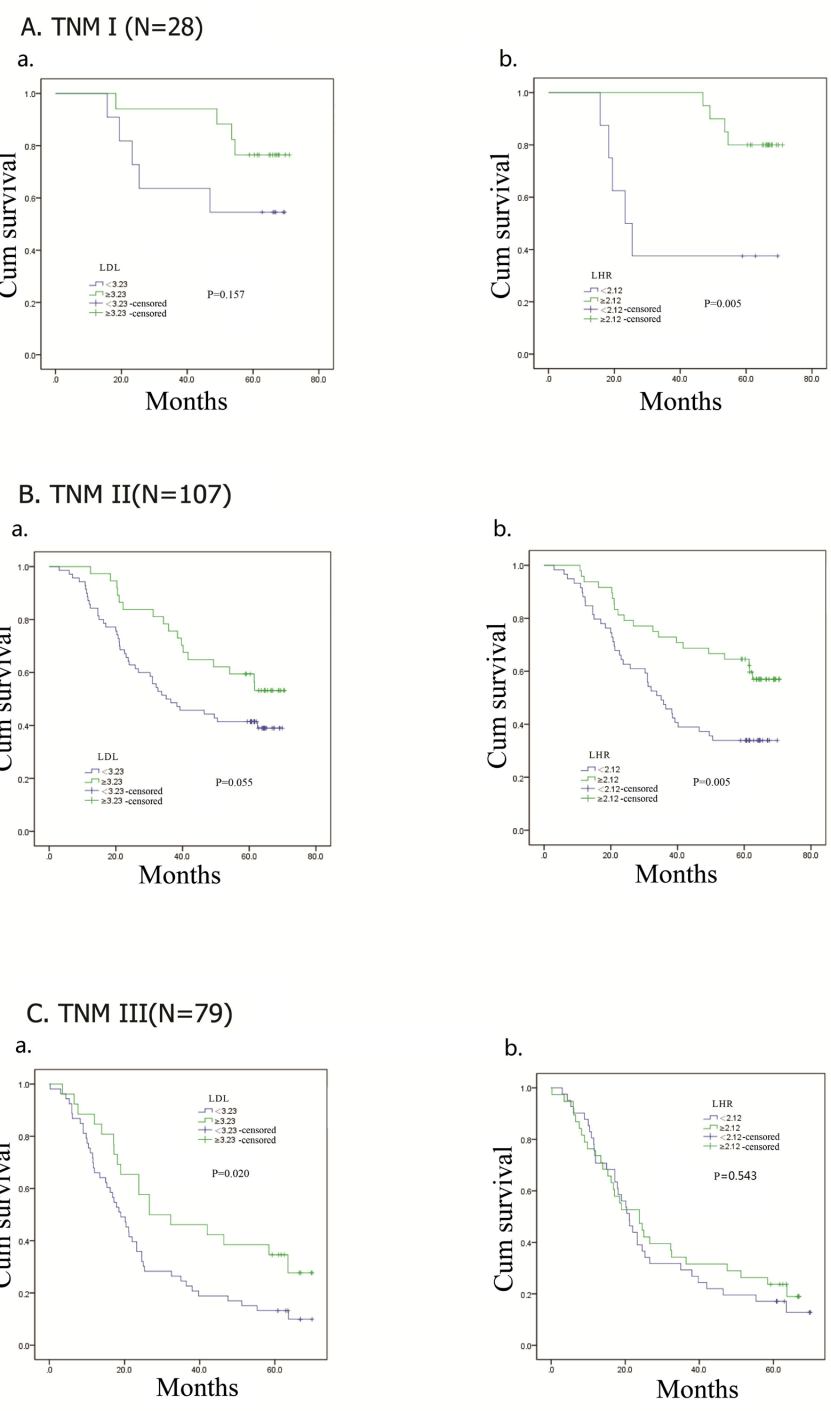
Kaplan–Meier analysis for overall survival of TNM I, II, III esophageal squamous cell carcinoma patients according to preoperative LDL-C, LDL-C/HDL-C ratio (LHR)

In the analysis of DFS (Figure 4), patients with high LDL-C had longer DFS in stage III (*p* = 0.023) group. No relationship was observed between LDL-C and DFS in stage I (*p* = 0.174) and II (*p* = 0.053) groups. High LHR had significant relationship with better DFS in patients with stage I (*p* = 0.005) and II (*p* = 0.003), but no positive outcome was found in stage III (*p* = 0.573) group. OS of stage III (*p* = 0.020) patients with high LDL-C was superior. In patients with stage I (*p* = 0.157) and II (*p* = 0.055) tumors, LDL-C had no relationship with OS (Figure 5). OS of patients with low LHR was significantly shorter when patients had stage I (*p* = 0.005) or II (*p* = 0.005) tumors, but not stage III (*p* = 0.543).

### The predictive effects of LDL-C and LHR for 1-year and 3-year OS

**Figure 6:**
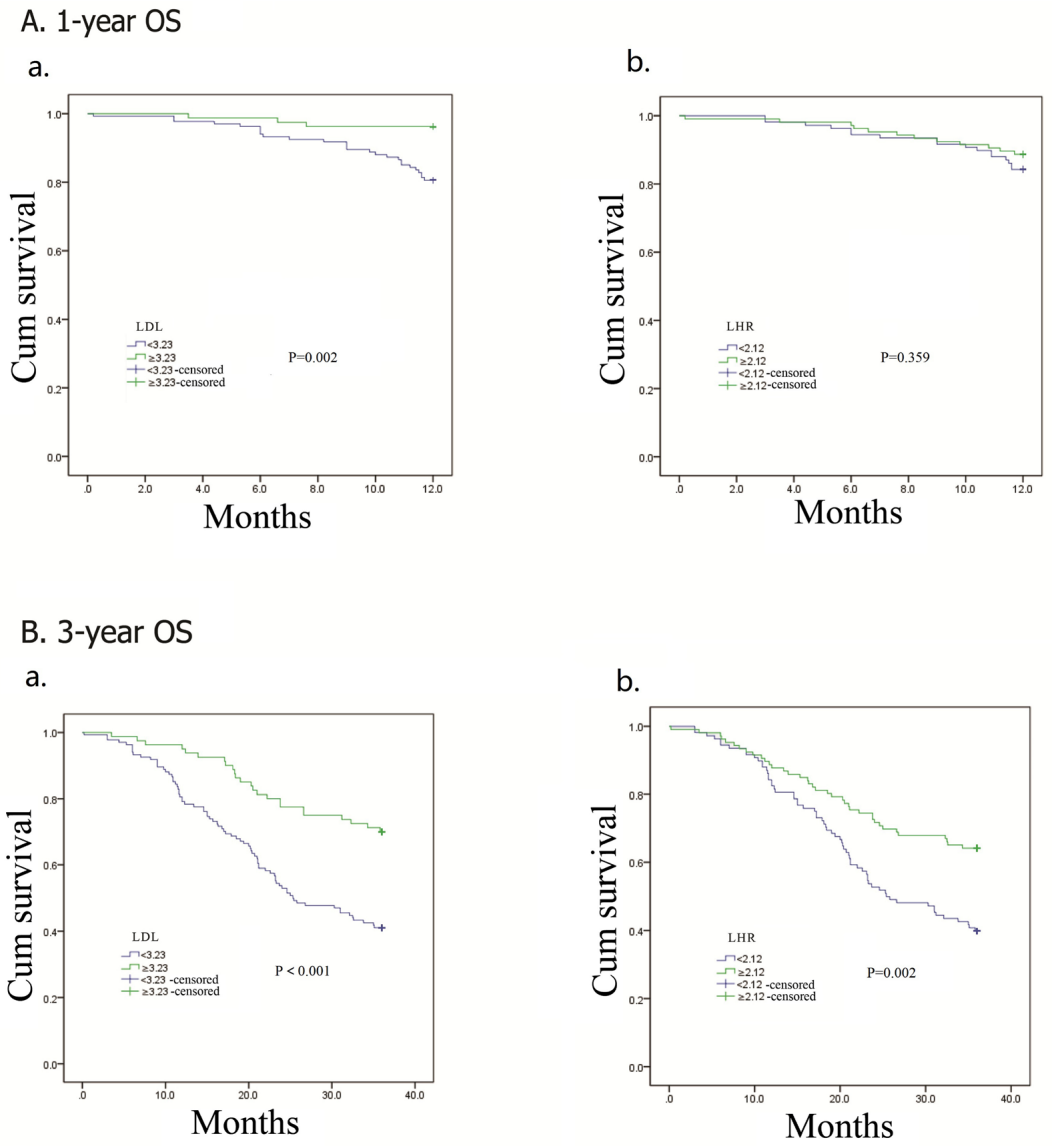
Kaplan–Meier analysis for 1-year and 3-year overall survival of 214 esophageal squamous cell carcinoma patients according to preoperative LDL-C, LDL-C/HDL-C ratio (LHR)

In this analysis, 1-year and 3-year OS were analyzed respectively (Figure 6). Patients with low LDL-C had inferior 1-year (*p* = 0.002) and 3-year (*p* < 0.001) OS. Statistical relationship between low LHR and poor OS occurred in 3-year OS (*p* = 0.002) analysis, but not 1-year OS (*p* = 0.359).

## DISCUSSION

In the present research, we evaluated the prognostic predictive values of preoperative serum lipids and the LDL-C/HDL-C-based LHR in ESCC patients who adopted esophageal resection. We found LDL-C and LHR were independent indicators for DFS and OS. Lower TC, LDL-C and LHR level could predict both poorer DFS and OS, especially the LDL-C and LHR. LDL-C and LHR owned different values for patients with different TNM stage and different survival time. These indicated that LDL-C and LHR could provide effective and important prognostic information in ESCC patients who underwent esophagectomy.

Cholesterol, as one of the structural component of cell membrane, is involved in some signaling pathways that are essential to malignant transformation [14]. LDL-C and HDL-C are key lipoproteins in the transportation of cholesterol. Studies had explored the relationship of cholesterol and LDL-C with cancer. It is reported that people with lower LDL-C level were more likely to generate tumor and most cancer-bearers got low LDL-C and TC levels [11, 15]. Saito et al suggested that lower LDL-C level occurred in liver cancer patients, and mortality was the highest in the lowest TC group [12]. Other researches also implied that low LDL-C and cholesterol were accompanied by obviously elevated cancer risk and mortality [16–19]. In the present study, we found the median DFS and OS were 18.3 and 25.4 months in low LDL-C group while 50.0 and 59.8 months in high LDL-C group (*p* < 0.001). The median DFS and OS were 18.8 and 31.4 months in low TC group, compared with 38.2 and 53.5 months in high TC group (for DFS, *p* = 0.031; for OS, *p* = 0.016). The mortality of patients with low LDL-C was 70.1%, which was 1.44 times higher than that in high LDL-C group (*p* = 0.002). Studies [8, 20] found that TC and LDL-C levels were obviously lower in patients with esophageal, gastric or breast cancer, even in early stage cancer-bearers. This suggested that the lipid changes might not be the products of reduced food intake or deteriorative nutritional status. We analyzed TC and LDL-C in patients with normal serum albumin and found the positive results.

Previous researches also supported our results. Firstly, tumor-associated hypocholesterolemia had been hypothesized as elevated LDL-C uptake in tumor cells, and TC and LDL-C would reduce because of the tumor consumption [8, 10]. As Y. Tomiki reported, we also found negative outcomes for HDL-C and TG implying low TC might stem from low LDL-C [8]. This might explain why TC exhibited slight statistical relationship with DFS and OS in our analyses. High pretreatment TC and LDL-C levels might represent low LDL-C uptake of tumor cells meaning low tumor metabolic activity. Secondly, increased LDL-C could enhance human immunological response and promote clearance of pathogen as reported [21]. Thirdly, other reports confirmed that oxidized low-density lipoprotein (oxLDL-C) had cytotoxicity on different cell lines and could activate apoptosis of esophageal cancer cells [22–24]. Fourthly, recent studies suggested that LDL-C could inhibit angiogenesis by disrupting hypoxia inducible factor (HIF) pathway and hindering endothelial cell migration [25, 26]. Angiogenesis could promote tumor development and showed association with tumor necrosis, which was a hallmark of poor prognosis [27–29]. Besides, a cohort suggested that higher LDL-C was associated with a lower mortality of all causes [30].

In previous studies [5, 31], LHR acted as a prognostic factor in colorectal cancer patients, providing different information from LDL-C. Our results indicated that ESCC patients with low LHR had poor survival outcomes. The median DFS in low and high LHR groups were 18.4 and 47.4 months (*p* < 0.001). The median OS of these two groups were 25.6 and 59.7 months (*p* < 0.001). To confirm whether LHR was merely affected by LDL-C or not, we stratified patients according to elevation ( ≥3.4mmol/L) of LDL-C. In patients with elevated LDL-C, the LHR had no relationship with DFS and OS. But DFS and OS showed statistical difference between two LHR groups in patients with no elevations in LDL-C. The recurrence rate was 54.6% in low LHR group, which was 1.38 times higher than that in high LHR group (*p* = 0.030). The recurrence rate had no statistical difference according to LDL-C. These suggested LHR could provide much prognostic information, even when preoperative LDL-C was normal. The mechanisms might be involved in the relation of dysfunctional lipid metabolism and cancer development [6, 8, 9].

Considerding the effects of adjuvant treatment on prognosis, patients were stratified according to different adjuvant treatment modalities. We observed that LDL-C and LHR were correlated to DFS and OS when patients had no adjuvant treatment or received adjuvant chemoradiotherapy. The findings confirmed the prognostic predictive values of LDL-C and LHR both in patients with or without adjuvant treatment after esophageal resection. Our subgroup analysis discussed the predictive functions of LDL-C and LHR in patients with different TNM stages and survival time. In Kaplan–Meier analysis, LDL-C level had statistical relation with DFS and OS in patients with stage III tumors. DFS and OS showed difference between two LHR groups in patients with stage I and II tumors. These suggested that LDL-C was more valuable for stage III patients, while LHR was better in stage I and II patients. LDL-C had close relationship with 1-year and 3-year OS, but no difference was found in two LHR groups regarding 1-year OS. The area under the ROC curve (AUC) was 0.636 (95% CI, 0.561-0.710; *p* = 0.001) for LDL-C and 0.622 (95% CI, 0.547-0.697; *p* = 0.002) for LHR in 3-year OS. As to 5-year OS, the AUC was 0.606 (95% CI, 0.528-0.685; *p* = 0.009) for LDL-C and 0.618 (95% CI, 0.541-0.695; *p* = 0.004) for LHR. These results implied that LHR had no prognostic effect on short-term survival, and LDL-C and LHR were associated with long-term survival.

There were some limitations in our research. First, it was a retrospective cohort with a small sample. Second, we did not figure out the causal relationship of low serum lipids and cancer development. It is hard to say whether low TC and LDL-C can promote cancer development or is the result of advanced cancer. Third, it was uncertain whether other cutoff values could act as better predictors. Fundamental knowledge is needed to elucidate the association of serum lipids and survival time in ESCC patients, and further basic and clinical researches are required.

In conclusion, low serum TC and LDL-C levels are predictive factors for poor prognosis in ESCC patients who underwent esophagectomy. LHR, as an independent factor, can serve as a novel and promising serum lipids-based prognostic indicator, especially for patients with earlier pathological stage. Measurements of serum lipids are inexpensive, convenient and routinely tested in clinic, especially before surgery. This will provide potential prognostic information for clinicians to facilitate the personalized treatment.

## MATERIALS AND METHODS

### Patients

During January 2007 to December 2008, 214 patients with esophageal squamous cell carcinoma who received esophagetomy in the Department of Thoracic Surgery, Qilu Hospital of Shandong University, were admitted into our retrospective study. A patient was excluded from the study: 1) if the patient had more than one primary tumors; 2) if the patient received neoadjuvant therapy; 3) if the patient had serious metabolic diseases before; 4) if the patient was lost during our follow-up. All the clinicopathological features came from patients’ records. The tumor was staged on the basis of the American Joint Committee on Cancer staging manual (seventh edition, 2010). The hematological and laboratory data were tested within one week before the surgery. And this study was ratified by the Ethics Committee of Qilu Hospital of Shandong University.

### Follow-up

Patients were followed up by telephone and face-to-face communication in the outpatient clinics every 3 months in the first 2 years after surgery. Then the patients’ information were obtained every 6 months until they were lost or dead. The ending time of follow-up was November 2013. Follow-up items included basic physical examination, laboratory blood or urine tests and routine computed tomography. The endpoints of our study were 5-year DFS and 5-year OS. DFS was defined as the time interval (in months) following by surgery during which patients lived with no tumor recurrence or advancement occurred. OS was the time interval (in months) from the date of surgery to death or the last follow-up date. In this study, we used the DFS and OS at the end of follow-up as the 5-year DFS and OS approximately.

### Serum lipids and LHR evaluation

Serum lipids levels, including triglyceride (TG), TC, LDL-C and HDL-C, were collected routinely from blood tests before surgery. The LHR, as a ratio, was calculated using dividing serum LDL-C value (mmol/L) by serum HDL-C value (mmol/L).

### Statistical analysis

Statistical analysis was performed using the Statistical Package for Social Science (SPSS for Windows, version 20.0, SPSS Inc., Chicago, IL) program. Serum TG, TC, LDL-C, HDL-C and LHR were shown as the mean and standard deviation (SD) and were assessed across the differential grade, T classification, N classification, pathological stage and recurrence. Correlations between categorical variables were evaluated by the Pearson's chi square test or Fisher's exact test in this study. And an unpaired *t* test and one-way analysis of variance were adopted in assessing continuous variables in different subgroups. The probable cutoff value levels for these continuous serum lipids and LHR were calculated by applying receiver operating curve (ROC) analysis. ROC curves were also adopted to verify the accuracy of LDL-C and LHR for survival prediction. In DFS and OS analyses, the Kaplan–Meier method and log-rank tests were used in this study. Besides, the Cox proportional hazards regression model was applied in our univariate and multivariate analyses. Those variables which exhibited statistical significance in the univariate analysis were included into the multivariable analysis. The two-sided *p* value was used in our analyses, and a *p* value of less than 0.05 represented statistical significance.
